# Groundwater implications on methane emission from non-sewered sanitation systems in Nepal☆

**DOI:** 10.1016/j.envpol.2024.124248

**Published:** 2024-05-27

**Authors:** Prativa Poudel, Prayon Joshi, Sarana Tuladhar, Anish Ghimire, Manish Baidya, Guy Howard, Subodh Sharma

**Affiliations:** a Department of Environment Science and Engineering, Kathmandu University, Nepal; b Aquatic Ecology Centre, School of Science, Kathmandu University, Nepal; c Youth Innovation Lab, Kathmandu, Nepal; d Department of Civil Engineering, Cabot Institute for the Environment, University of Bristol, UK; e Environmental Engineering and Management Program, Department of Energy, Environment and Climate Change, Asian Institute of Technology, Pathum Thani, 12120 Thailand

**Keywords:** Groundwater, Pit latrine, Septic tank, Non-sewered, Greenhouse gases, Methane

## Abstract

Non-sewered sanitation systems (NSSS) are identified as significant contributors of greenhouse gases (GHGs), primarily due to biological processes within the containment systems. In unsealed or unlined containment systems like pit latrines, the emissions are influenced by moisture. This work quantified the GHG emission occurring from unlined or unsealed containments prevalent in Nepal and compared it with sealed containment-like septic tanks, where the chances of groundwater (GW) inundation are low. The modeled GW data extracted from the secondary sources were validated with available national data. The emissions were quantified using the Intergovernmental Panel for Climate Change (IPCC) model for different ecological divisions and provincial divisions of Nepal. Spatial representation for the results was done using the Geographical Information System (GIS) tool. The total methane (CH_4_) emission occurring from the various NSSS was determined to be 2618 Gg CO_2_ e per year which is almost twice the emission from the waste sector, as reported by the recent national communication submitted to the United Nations Framework Convention on Climate Change (UNFCC). Variation of the CH_4_ emission was found to be prominent in lowlands (Terai region) with total national emissions of 1329.37 Gg CO_2_e per year. The lowland has a shallow GW table that can easily inundate the unlined containments like pit latrines thus contributing to more anaerobic conditions which may lead to higher CH_4_ emissions compared to containments in mid and highlands. This study concludes that the GHG emissions occurring from NSSS are substantial and addressing these emissions can help fulfil the Nationally Determined Contributions (NDCs) in the waste sector.

## 1. Introduction

Non-sewered sanitation systems (NSSS) which include containments such as unlined or unsealed pit latrines and sealed septic tanks are widely adopted in managing fecal sludge (FS) in low- and middle-income countries. Around 43% of the global population relies on NSSS for safe FS disposal and the preference for this has grown rapidly compared to sewer systems, due to their acceptability and affordability ((World Health Organization and the United Nations Children’s Fund, 2021). Development and increased use of NSSS technologies are ever-growing but the basic concept and biological process involving the anaerobic and aerobic digestion inside any containment systems remains unchanged (Van Eekert et al., 2019). The anaerobic process includes the breakdown of complex organic matter through several mechanisms including fermentation, acidogenesis, acetogenesis, and methanogenesis. These processes partially digest FS that consists of carbohydrates, proteins, and lipids in the presence of several interacting anaerobic microorganisms resulting in the emission of greenhouse gas (GHG) such as methane (CH_4_) and carbon dioxide (CO_2_) along with a few soluble byproducts (Sanders, 2001). This phenomenon could be observed in septic tanks and pit latrines. Along with the anaerobic processes, oxidation of the organic matter in the presence of oxygen helps form CO_2_ in the pits and soil dispersion units (Leverenz et al., 2010). This process is often termed as an aerobic process. However, the findings suggest that the exact process occurring inside the various containment systems is not clear yet and remains uncertain. Nevertheless, some studies have shown that both kinds of processes occur inside the pits, with the aerobic process occurring at the top layer and the anaerobic process at the bottom (Bourgault et al., 2019). Other studies have found that aerobic processes are dominant process in unsealed containments (Chaggu, 2004; Van Eekert et al., 2019). Additionally, the process inside such unsealed pit latrines are also dependent on the design and ventilation of the pits (Buckley et al., 2008). Variation in the process inside any containment system results in differences in the emission of the GHGs. The estimation and reliable reporting of GHGs from the containment systems is important in the present context of global warming and mitigation pledges by countries as the contribution of such gases as the GHG emissions from these NSSS is considerable.

Studies have made it evident that the GHG emissions from sanitation systems are occurring continuously but are often overlooked and have a detrimental impact once they start escaping to the environment (Calvin et al., 2023). A recent comprehensive meta-analysis on NSSS revealed that globally, methane production of nearly 377 Mt CO_2_ e per year of which septic tanks account for 55.97% of emissions, while pit latrines contribute the remaining (Cheng et al., 2022). With similar findings, Reid et al. (2014) estimated the global emissions occurring from pit latrines to be 2% in 2000. With the increased number of NSSS like pits in the global south, an increase in GHG emissions is also expected (Reid et al., 2014). These emissions are often governed by factors such as moisture content (Van Eekert et al., 2019). The sources of moisture in NSSS come from groundwater inundation and any liquid introduced through anal cleansing and urine. Of these, groundwater is anticipated to be the largest component in most cases (Reddy et al., 2023). The effect of groundwater inundation is normally seen in systems with semi-sealed or unsealed containments which is a typical characteristic shared by pit latrines (Reid et al., 2014). An increasing level of moisture content enhances the mass transfer, enabling the efficient diffusion of substrates to microorganisms and the movement of products away from them. This, in turn, promotes an improved rate of biodegradation, thus producing a high amount of GHGs (Martin et al., 2003). These experimental findings are supported by some studies which have shown inundated pits are associated with higher emission rates compared to dry pits (Reddy et al., 2023; Reid et al., 2014; Ryals et al., 2019).

The Intergovernmental Panel for Climate Change (IPCC) have set out standard modeling based methods for calculating GHGs at national levels from different sectors (Bartram et al., 2019). The IPCC guidelines provide different emission factors (EFs) for various containment systems depending upon wet and dry conditions within the NSSS. The methane conversion factor (MCF) and EF recommended by IPCC for septic tanks and pit latrines in a dry climate with multiple users are 0.5 and 0.3 kg CH_4_ per kg biological oxygen demand (BOD), respectively whereas in wet climates it is 0.7 and 0.42 kg CH_4_ per kg BOD (Bartram et al., 2019). Although, IPCC note that hydrological control over CH_4_ emissions is important, there are very few studies that have discussed and evaluated the influence of hydrogeology over CH_4_ emissions (Reddy et al., 2023; Reid et al., 2014).

Though the total global emission contributions from Nepal are around 0.027 %, it is one of the most vulnerable countries in terms of the impacts of climate change (Ministry of Population and Environement Government of Nepal, 2016). In a second nationally determined contribution (NDC) reports, Nepal aims to reduce the significant amount of GHG emissions from different sectors including wastewater (GoN, 2020). Nepal aims to reduce 258 Gg CO_2_e per year of the total emissions occurring from the waste sector by treating 380 million liters per day of wastewater and managing 60,000 m^3^ per year of FS (GoN, 2020). An accurate estimation and reporting is equally important, to meet the target set by the NDCs report.

Sanitation systems in Nepal are distributed across all the geographical areas with its major concentration in low-land areas due to dense population (CBS, 2021). Septic tanks and pit latrines are two major types of sanitation technologies used in Nepal. While the septic tanks are designed to be sealed, although this may not be the case in reality, pit latrines are permeable to the moisture from GW at the subsurface level. In this study, we have categorized NSSS into 3 categories: septic tanks, dry unsealed/pit latrines, and wet unsealed/pit latrines. GW is found in almost all places but the level varies according to the geomorphological variations. Previous global studies have indicated that the typical depth of the containments for pit latrines varies from 1 to 3 m, which increases the risk of GW inundation and surface water flooding, especially in lowlands (Cotton et al., 1995). Although the IPCC provides different EFs dependent on moisture conditions, due to the limited availability of reliable national GW data, quantification is often based on a single EF for both wet and dry conditions of the containment.

A comprehensive estimation of such emissions influenced by GW has been lacking in Nepal. While a scenario-based study by Shrestha et al. (2022) investigated this issue, it did not explicitly consider the impact of GW (Shrestha et al., 2022). Thus this study aims to fill the gap by being the first to assess GHG emissions from Nepal’s NSSS integrating the influence of GW on containment conditions and its implications for methane emissions. The findings of this work can serve as a valuable reference low and middle-income countries with the challenge of quantifying such emissions in relation to environmental factors like GW. Moreover, for countries like Nepal with difficult terrain, the measurement of the emission from a large number of sources takes up a lot of resources.

In this study, data from a national-level survey conducted in 2021 is used, which include the most recent datasets for the NSSS technologies which are currently being used at the household levels (CBS, 2021). This data includes the population fraction using different non sewered containment technologies in different parts of Nepal (CBS, 2021).

## 2. Materials and methods

This study was carried out using the method outlined in “Chapter 6: Wastewater Treatment and Discharge” of 2019 Refinement of the 2006 IPCC Guidelines for National Greenhouse Gas Inventories (Bartram et al., 2019). This guideline suggests three Tiers of calculation for the determination of National GHG contributions. Given the unavailability of country-specific data for the wastewater sector in Nepal, this study employed the Tier 1 method to report emissions from different NSSS. This specific study only considers CH_4_ emissions from the containment system. CO_2_ and N_2_O were not considered for the calculations of the total GHG. CO_2_ is considered a biogenic emission by the IPCC. Unlike CO_2_, N_2_O emissions are dependent on very dynamic and complex processes and are not linked to GW inundation by IPCC emissions.

### 2.1. MCF and EF

The MCF is the fraction of organic matter that is converted into CH_4_ during the anaerobic decomposition process within the containment system (Bartram et al., 2019). GW inundation can significantly affect this process which is usually observed in pit latrines due to its unsealed characteristics (Reddy et al., 2023). A GW table higher than the pit depth can cause the pit latrine anaerobic, affecting MCF values. In our study, we considered the pit depth threshold of 3 m (m) using the maximum typical depth as described by Cotton et al. (1995). This threshold level is in line with previous studies (Reid et al. 2014; Reddy et al. (2023) who used the cutoff height as 2.5 ± 0.5 m. In the case of Nepal, the recommended effective height of the pit latrine is 3 m, (SNV, 2010). Various MCF and EF values for wet pit latrines, dry pit latrines and septic tanks were selected for CH_4_ estimations (Table 1).

For septic tanks, a constant MCF value of 0.5 is used, assuming that these systems are sealed and there is no GW inundation in such systems (Bartram et al., 2019). Similarly, the maximum methane producing capacity (B_o_) for Nepal was 0.6 kg CH_4_ per kg BOD for domestic wastewater that was used to calculate the emission factor (EF) (in kg CH_4_ per kg BOD) represented by equation 1:

(equation 1) EF=Bo*MCF

### 2.2. CH_4_ emissions calculations

CH_4_ emission from domestic wastewater for each containment i.e., septic tank or pit latrine calculated as per equation (2). The default values used for determining the emission are tabulated in Table 2.

(equation 2) $${\text{CH}}_{4}\;\text{Emission}\;(\text{kg}\;{\text{CH}}_{4}\text{per year})\;=\left[\left({\text{TOW}}_{\text{j}}-{\text{S}}_{\text{j}}\right)\times {\text{EF}}_{\text{j}}-{\text{R}}_{\text{j}}\right]$$

$TOW_{j}=$ organics in wastewater system in inventory year, kg BOD_5_ per year. Biochemical oxygen demand (BOD) is the amount of dissolved oxygen consumed by microorganisms in the biochemical oxidation of organic and inorganic matter in wastewater. The BOD-5 represents the level of oxygen demand during the 5 days of incubation at 20 °C.

$S_{j}$ = organic component removed from wastewater in inventory year (kg BOD_5_ per year)

j = treatment/discharge pathway or system

$EF_{j}$ = emission factor for treatment/discharge pathway or system (kg CH_4_ per kg BOD_5_).

$R_{j}$ = amount of CH_4_ recovered or flared from treatment/discharge pathway or system, j, in inventory year (kg CH_4_ per year), the default value is zero.

Total organics in domestic wastewater by treatment pathway is calculated as in equation (3):

(equation 3) $$TOW_{j}=\sum \left[TOW\times U_{j}\times T_{ij}\times I_{j}\right]$$

TOW = total organics in wastewater in inventory year, kg BOD_5_ per year.

$U_{j}$ = fraction of population in income group i in inventory year.

$T_{ij}$ = degree of utilization of treatment/discharge pathway or system, j, for each income group fraction

$I_{j}$ = correction factor for additional industrial BOD_5_ discharged into treatment/discharge pathway or system j.

The fraction of urbanization (U_i_) and utilization ratio (T_ij_) were calculated using national census conducted in 2021 made available by the Central Bureau of Statistics (CBS), Government of Nepal (equations 4 and 5). Due to the unavailability of the country-specific economic classification data, sub-classification of economic division as high income and low income as instructed in 2019 Refinement of IPCC was not included in this study.

The U_i_ was calculated using equation 4:

(equation 4) $$U_{i}=\text{Population of different income levels}/\text{Total population}.$$

Furthermore, the value $T_{ij}$ of treatment system was also obtained from the census data using equation 5:

(equation 5) $$T_{ij}=\text{Population using a sanitation systems}/\text{Total population}.$$

Total organically degradable material in domestic wastewater is calculated as equation (6):

(equation 6) TOW=P×BOD×0.001×365

*TOW* = total organics in wastewater in inventory year, (kg BOD_5_ per year).

*P* = country population in inventory year, (person)

*BOD* = country-specific per capita BOD in inventory year, (g per person per day).

0.001 = conversion from grams BOD to kg BOD

The global warming potential (GWP) used for conversing the units to CO_2_ equivalent is 28 for 100-year time horizon.

Total emissions from NSSS, pit latrine and septic tanks are calculated using equations (7)–(9)

(equation 7) $$Total\;Emission=Emission_{pit\_latrine}\;+Emission_{septic\_tanks}$$

(equation 8) $$Emission_{pit\_latrine}=TOW_{pit\_latrine}\;*Emission\;factor_{pit\_latrine}$$

(equation 9) $$Emission_{septic\_tank}=TOW_{septic\_tank}\;*Emission\;factor_{septic\_tank}$$

With the utilization of the information presented in Tables 1 and 2, a comprehensive assessment of the TOW from the pit latrines and septic tanks was done in the local levels i.e. all 753 units. Total country population and municipal level population data were acquired from the National Census database (CBS, 2021). Computation of TOW values involved a systematic series of computations based on the parameters that were collected at household levels during recent census (<2 years). The GW table plays a vital role in determining whether the sanitation system is in wet conditions or dry conditions which will further affect the emission factor of the sanitation system. Thus, to bring the GW table into the picture together with the TOW values, a sequence of techniques was deployed for extrapolating the municipal-level TOW values into the spatial grid-cell framework.

Variation in the ecological zones of Nepal stems from the contrast in the topography. The country ranges from 64 masl in the southern plain Terai Plain area (lowland) to the 8848 masl in the higher Himalayan region (highland). In the lowlands of Nepal, the GW is abundantly available with high a recharge rate of 8800 MCM/year (Shrestha et al., 2018) resulting in a higher GW table and occurrence of moist conditions in containments for most of the year and especially during the monsoon. In this study, for each local unit, i.e. municipality, a centroid was selected. Getting the centroids of the polygons acted as a locational anchor, with the centroids the polygon data of municipal boundaries were converted to point data. Further, the points were assigned with the TOW values which were associated with the Septic Tanks and Pit Latrines of the municipal units. 753 points with TOW values were obtained which further needed to go through the interpolation to obtain the gridded values for Nepal. The interpolation of TOW values with the spatial domain was done with the Inverse Distance Weightage (IDW) method. The weights are inversely related to the distances between the sampled and predicted locations (Lu & Wong, 2008).

IDW is a deterministic model which has been previously used in studies for the interpolation of emission values with similar areas as ours (Reddy et al., 2023). This technique is relatively efficient to compute and straightforward to interpret making it the most used interpolation technique. This technique is used to estimate values of the unsampled points from the values from previously known points, and the estimation is done assuming that the weights are inversely related to distances between the known points and the unsampled locations (Lu & Wong, 2008). Mathematically, if the value of the unsampled location is considered $Z\left(x_{0}\right)$, which lies at a distance of x, with a linear combination of weights $\lambda_{i}$ and the values of sampled location is Z as shown in equation (10).

(equation 10) $${\text{Z}}^{\text{*}}\left(\chi_{0}\right)=\sum \lambda_{\text{i}}\text{Z}\left({\text{x}}_{\text{i}}\right)$$

The value of $\lambda_{i}$ is represented by equation (11).

(equation 11) $$\text{Where},\lambda_{i}\frac{{d_{0}^{-a}}_{i}}{\sum_{i=1}^{n}{d_{0}^{-a}}_{i}}$$

The summing of individual weights (λ), results as 1, as shown in equation 12

(equation 12) $$\sum_{i=1}^{n}\lambda_{i}=1$$

The value of Z at the point x_0_ is a weighted sum of Z values at other points where each Z value is multiplied by a corresponding weight λ. Whereas, the λ i.e. weightage of a particular point is determined by taking the reciprocal of the distance raised to the negative power of α. Finally, the α is the weighting parameter which controls how the weight drops off as the distance increases. Summing up, the closer the unknown point from the known point higher the values and vice versa. For this research, we have used default weightage for interpolation present QGIS software (version 3.28 LTS).

This technique generates meaningful results even in regions with limited data points, thus this technique was considered for the interpolation of total CH_4_ emissions calculations for each containment type using different EFs and spatially represented through a map.

### 2.3. Validations and error analysis

We checked the accuracy of their modeled GW by comparing it with existing national GW data. Modeled emission results were compared with a dataset generated by the Sanitation and Climate: Assessing Resilience and Emissions (SCARE) project (result not shown).

For this, two types of error analysis were carried out to estimate mean absolute error and bias. The mean absolute error showed an overall measure of prediction of accuracy while the bias showed if the values are overestimated or underestimated. Bias was calculated as an average of the difference in the values of modeled and observed datasets. The absolute error percentage was calculated using the following equation (13):

(equation 13) $$Error\;percentage=\frac{modeled-observed\;value}{observed\;value}$$

## 3. Results and discussion

### 3.1. GW table variation within Nepal

**Fig. 1.**
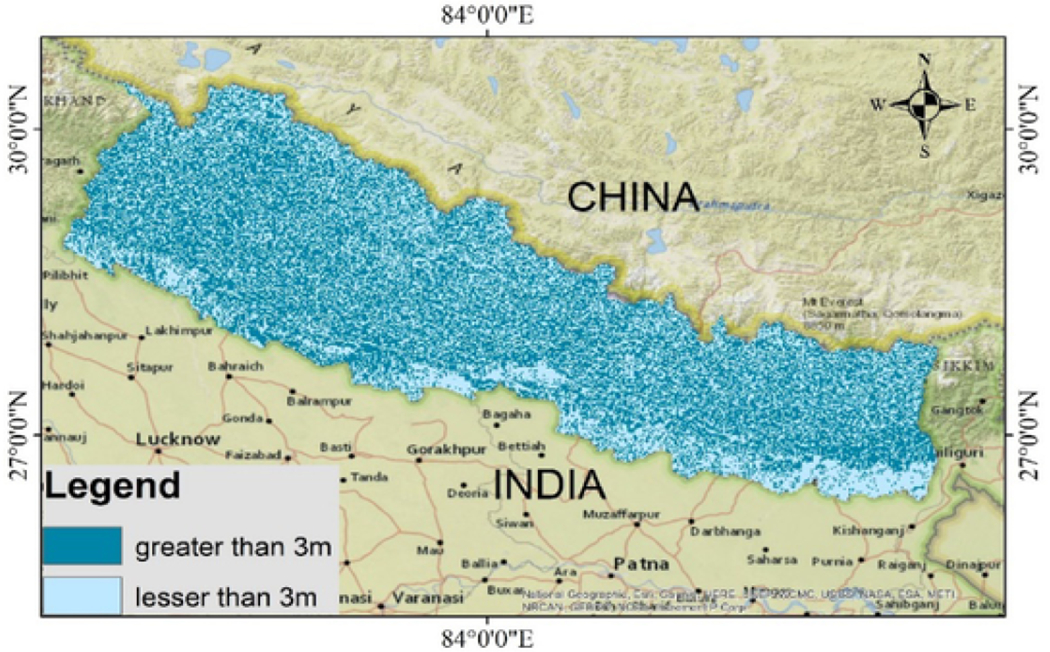
GW table variation throughout the Nepal, light blue representing the deep groundwater table and dark blue representing the shallow GW table. (For interpretation of the references to color in this figure legend, the reader is referred to the Web version of this article.)

The GW-level observations were adapted using the database from Fan et al. (2013). The available national GW data are very limited and cannot be used to estimate water levels across the country. Fan et al. (2013) produced a global pattern of GW depth using a GW model considering the modern climate, terrain, and sea level (Fan et al., 2013). Considering the various geographical categorization of the country, variation of the GW table was observed between the eco-regions. Nepal is divided into three ecoregions – Lowland, Midland and Highland. The lowlands with higher GW tables which are generally less than 3 m and the midland and highland with lower GW table with depths greater than 3 m (Fig. 1).

**Fig. 2.**
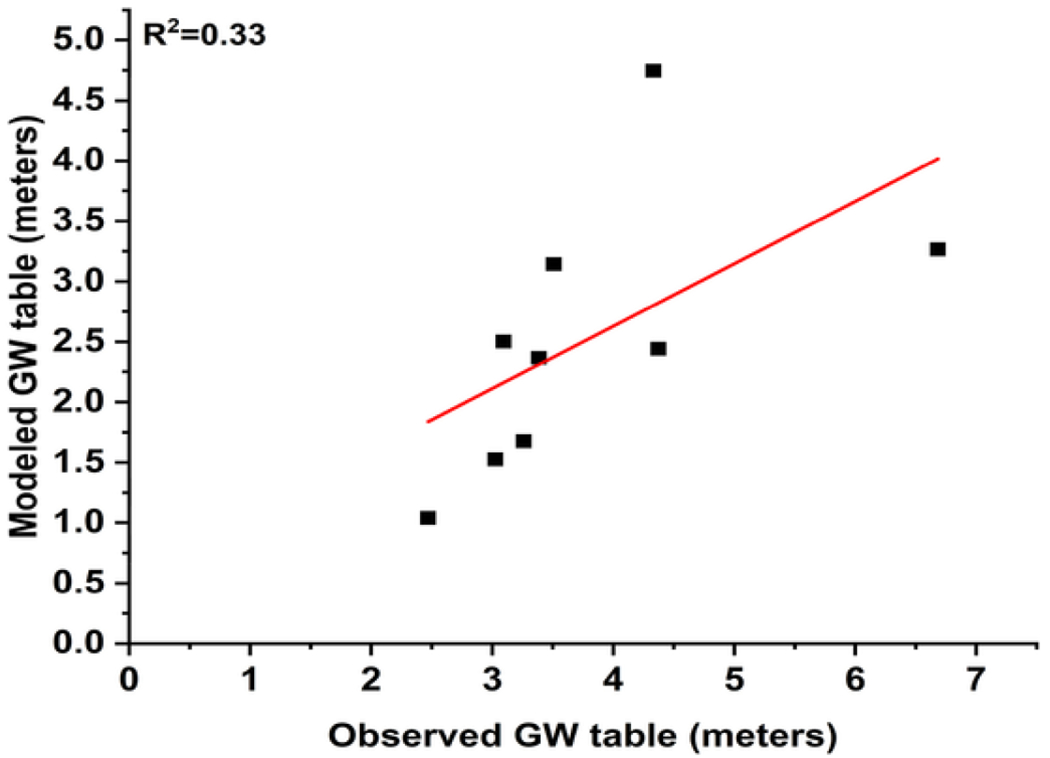
Linear plot between observed GW table and modeled GW table in meters.

Modeled GW table extracted from Fan et al. (2013) was validated using the data from the Ministry of Water Resources Energy and Irrigation (https://gw-nepal.com/). A total of 10 GW table points that were regularly monitored by the GW board were used to validate the modeled data. Overall, a negative bias of 0.8 m was observed. This denotes that the GW table value provided by Fan et al. (2013) to be lower than the observed value. The absolute mean error calculated between the modeled and actual GW table is 22%. Due to the unavailability of the GW data in highlands we could not validate the other data on GW table which remains a limitation to this study. The modeled and actual values fitted for GW had accuracy of 77.9 %. Observed and modeled emissions were linearly fitted to see the variation (Fig. 2). The value of $R^{2}$ indicates that the GW data has 33% variability around the mean. This suggests the model can be useful in areas with scarce GW information, although some error is expected.

### 3.2. Total GHG emissions from various containment types

**Fig. 3.**
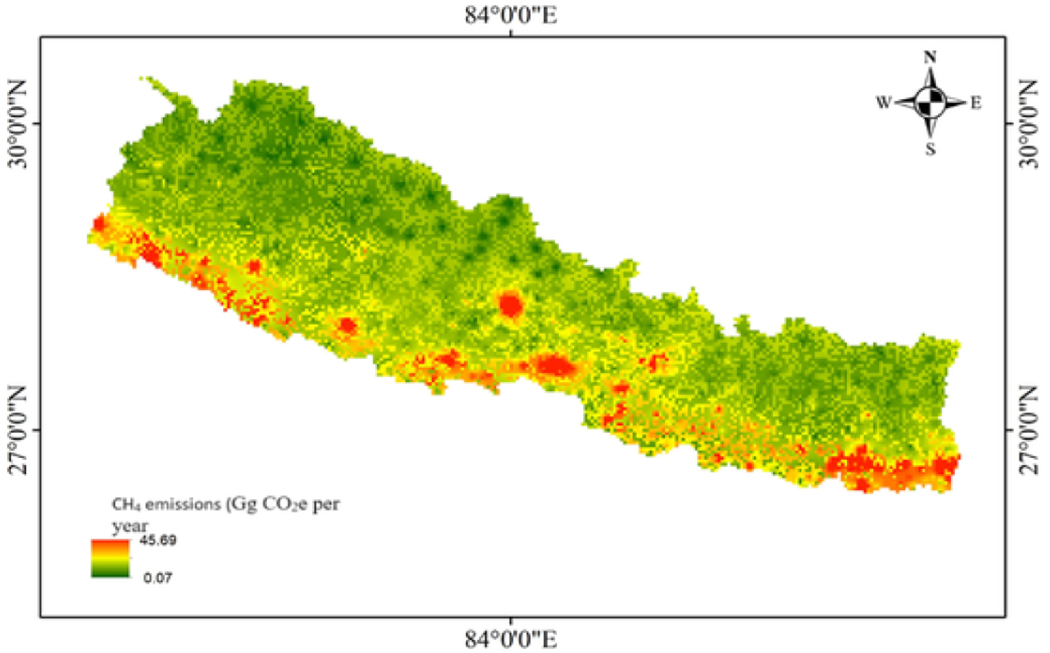
Geospatial emissions occurring from NSSS in Nepal. Red indicates higher emissions and green indicates the lower CH_4_ emissions. (For interpretation of the references to color in this figure legend, the reader is referred to the Web version of this article.)

The spatial representation of the emissions occurring throughout the country is depicted in Fig. 3. The total emission calculated from septic tanks is 1725 Gg CO_2_e year and that for pit latrines is 893.434 Gg CO_2_e per year. This is the first-ever result that reports the emission occurring from the septic tanks and pit latrine with implications of the GW in Nepal. Comparing this finding with the national emissions estimated from 2021, the current emissions from onsite sanitation are almost double occurring from the waste sector i.e. 923.5 Gg CO_2_e per year. The emission calculated from this study accounts for 9% of the total national emissions. It should be noted that the comparison of this result is made with the national estimate that has used the base year 2014. Similar to our study, Shrestha et al. (2022) calculated the emissions of from domestic wastewater of 3829.46 Gg CO_2_e per year for Nepal. This emissions accounts to 13.4 % of the total emissions of the country (Shrestha et al., 2022). However, the author has includedd various treatment process like activated sludge , waste stabilisation pond, and untreated sewers.

National statistics indicate that a similar number of the rural population use septic tanks as their urban counterparts, indicating current estimates substantially under-estimate emissions. Furthermore, the majority of septic tanks in Nepal are not designed properly, with estimates that more than 95% of contaminants claimed as septic tanks are modified pit or holding tanks (Shrestha, 2020). With similar findings, a situational assessment of fecal sludge management done in Mahalaxmi Municipality showed that only 12% of the reported septic tanks were proper septic tanks, while, others were intermittent tanks (ENPHO, 2019). This suggests our estimates are likely to be underestimates because assumed septic tanks as sealed when they are not and thus, likely to be inundated.

The result obtained from this study was compared to other studies that have predicted or calculated the emissions occurring from containments such as pit latrines with reference to GW table (Table 3). The total CH_4_ emissions that this study calculated for pits are within the range predicted by Reid et al. (2014) for Bangladesh, India and China for the year 2015(Table 3). The Reid et al. (2014) used an explicit spatial approach to determine the effect of local hydrology on CH_4_ emissions from pit latrines. The calculated total emissions of the world accounted around 3.8 Tg per year, which accounts around 1% of the total anthropogenic emissions. However, Reid et al. (2014) did not take into account of the seasonal variation of the GW table. Reddy et al. (2023) used seasonal variation of GW using GRACE model to calculate the emission occurring from the pit latrines in Senegal which was 568 Gg CO_2_ e per year (Reddy et al., 2023). A whole sanitation system analysis done in Kampala shows that the emission from anaerobic sealed tanks emits 49% of the emissions occurring from the whole sanitation value chain. The study in Kampala suggested emissions from sanitation were equivalent to 189 Gg CO_2_e per year GHG (Johnson et al., 2022). A scenario-based analysis that compared and predicted the emission from domestic wastewater with the advancement of technology showed that the emission in 2020 for Nepal to be around 3829.43 Gg CO2e per year (Shrestha et al., 2022) (Table 3). Estimated emissions for Nepal from Shrestha et al. (2022) is seven folds higher than the National estimates for domestic wastewater (532.7304 Gg CO_2_ e per year). However, the study done by Shrestha et al. (2022) did not take in account the effect of the GW table on containment conditions rather it was focused on different developmental scenarios in sanitation sectors.

The IPCC classification of pit latrines as “wet” or “dry” based on GW is a useful classification for accurate estimation of CH_4_. Wet pits or containment with high GW levels create anaerobic environments for CH_4_ production, while dry ones with lower GW do not (Reid et al., 2014). However, this classification does not consider other factors that can significantly impact emissions like the design of the containment, and temperature. For example, a well-sealed pit can reduce the influence of GW in CH_4_ production, regardless of GW level (Reid et al., 2014). Warmer temperatures and the specific properties of the FS in the pit (like chemical oxygen demand, oxidation and reduction potential) influence the type of process occurring and the rate of decomposition, further affecting CH_4_ production (Diaz-Valbuena et al., 2011; Huynh et al., 2021). A more comprehensive approach that takes into account environmental factors like temperature and rainfall, along with the FS characteristics is required to ascertain the actual conditions of the containments. This will allow us to develop better strategies to reduce methane emissions from sanitation systems and contribute to mitigating climate change.

### 3.3. Variation of emissions within the country

**Fig. 4.**
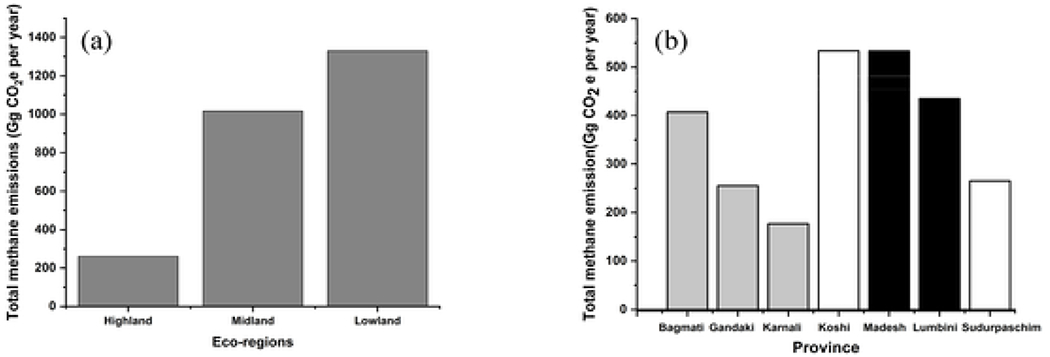
Greenhouse gases emission from NSSS in each (a) ecological regions and (b) seven provinces in Nepal. The provinces itself can be categorized as per ecological regions it lies. The black bar charts represent the coverage of the province in lowlands. White color bar chart represents the provinces that covers all the three ecoregions. Grey color represents the coverage of midland and highlands only.

Variation of the emissions based on three ecological divisions and seven administrative divisions was analysed. The calculated emissions are found to be high in lowland region (1329.36 Gg CO_2_e per year) followed by midland (1016.19 Gg CO_2_e per year) and highland (260.89 Gg CO_2_e per year) (Fig. 4 a). This variation in the CH_4_ emission can be related to GW table variations and the population density in each ecoregion. Lowlands of Nepal have a higher GW table compared to highland. Shrestha et al. (2018) identified lowlands as one of the potential locations for accessing GW. The GW table in lowlands varies significantly, ranging from 1.5 m to 18 m (Shrestha et al., 2018). This variation in depth suggests a high risk of flooding containments. Adding on to this the lowlands are more densely populated compared to other regions. The lowland has the highest population density 460 people per sq. km followed by midland and highland with a population density of 192 people per sq. km and 34 people per sq. km (CBS, 2021).

### 3.4. Validation of modeled emission

**Fig. 5.**
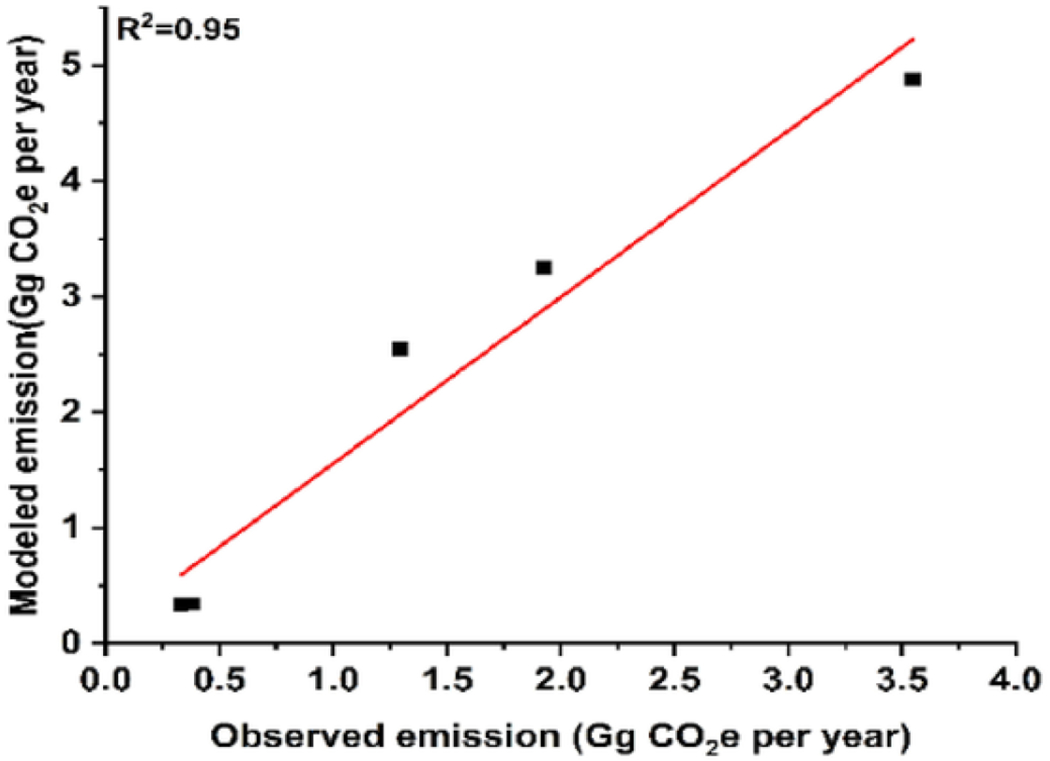
Linear plot between modeled emissions and observed emissions.

Validation for the emission obtained from this study was done using GHG emissions from the field measurement (full data not reported here) (Fig. 5). The method and field-based data used for validation were reported by the Sanitation and Climate Change Assessing Resilience and Emission (SCARE) project funded by the Bill & Melinda Gates Foundation (grant No: INV-015713) (results not shown). The modeled and actual values fitted for emission had an accuracy of 61.35%(Fig. 5). The absolute mean error between the data set ranged from 1 to 97%. The overall bias on average for both pit latrines and septic tanks was 0.77 Gg CO_2_e per year. The bias was positive, which clearly shows the emission estimated from the model is higher than the field-based measurements. Certain errors in estimations may be due to variation between the actual GW table and the modeled GW table.

GW table for Nepal used in this study has been extracted from a modeled global dataset. It is important to note that GW level is relative to season, however, this study does not incorporate seasonality as a factor of influence in the GHG emissions. Seasonal variation of the emissions is explained and elaborated by Reddy et al. (2023), which used the GRACE Model to show the variations (Reddy et al., 2023). Also, this study has not considered other factors that might play important roles like emptying frequency, management option, or the household type of cleaning material that can play role in CH_4_ emission, which remains another limitation to the study.

IPCC methods for estimation of GHG from various sectors are good where there are no country-specific datasets. Using country-specific data for MCF and EF leads to more precise calculations of GHG emissions. Field-based measurements can provide this data, that can fit to IPCC method for providing the better estimates (Poudel et al., 2023).

## 4. Conclusion

This study provides an estimation of GHG emissions from different common types of containments like pit latrines and septic tank in Nepal, which were found to contribute around 9% to the country’s total GHG emissions. GW table level can play significant role in emission occurring from unsealed containments of the country. Thus, consideration of GW factor is important in calculating the overall emission from sanitation sector. It is observed that current estimates included within national communication report grossly underestimate actual levels of GHG being emitted by pit latrines and septic tanks. The national estimates only have done the crude calculation using single emissions factors for both wet and dry containment type. However, other factor like GW inundation are important to categories the containment type that alters the EF and hence the GHG emissions. In addition to this, a more robust, detailed, and comprehensive national sanitation database is required to improve GHG inventory reporting for the waste sector which is only possible with better understanding of different sector including waste.

## Figures and Tables

**Table 1:** Default MCF and EF used in the calculations suggested by IPCC refinement (Bartram et al., 2019)

Type of treatment system	Comments	MCF	EF (kg CH_4_ per kg BOD)
Pit Latrines	Dry climate, GW table lower than latrine, small family (three to five persons)	0.1	0.06
Pit Latrines	Wet climate/flush water use, GW table higher than latrine	0.7	0.42
Septic tanks	Impermeable to surrounding moisture/GW	0.5	0.3

**Table 2:** Parameters used for calculation of CH_4_ emissions from the septic tanks and pit latrines for year 2011 and 2021.

Parameters	Values
S_j_	0
R_j_	0
BOD for Asia	40
Ij	1

**Table 3:** Comparison of the total GHG emission from different countries reported by previous studies.

Author(s)	Country or City	Containment type	Total emission (Gg CO_2_e per year)
Reddy et al. (2023)	Senegal	Pit	568
Reid et al. (2014)	Bangladesh	Pit	443–1114.0
Reid et al. (2014)	China	Pit	751–1983
Reid et al. (2014)	India	Pit	179–700
Johnson et al. (2022)	Kampala (Uganda)	Whole sanitation value chain	189
Shrestha et al. (2022)	Nepal	Domestic wastewater	3829.43
**Our Study**	Nepal	Septic tank	1725
**Our Study**	Nepal	Pit latrine	893.43
**Our Study**	Nepal	NSSS	2618

## Data Availability

Data will be made available on request.
